# Growth, seed development and genetic analysis in wild type and *Def *mutant of *Pisum sativum *L

**DOI:** 10.1186/1756-0500-4-489

**Published:** 2011-11-11

**Authors:** Kwadwo Owusu Ayeh, YeonKyeong Lee, Mike J Ambrose, Anne Kathrine Hvoslef-Eide

**Affiliations:** 1 The Biotechnology and Nuclear Agriculture Research Institute (BNARI), Ghana Atomic Energy Commission (GAEC), P.O.Box Lg. 80, Legon-Accra, Ghana; 2 Department of Plant and Environmental Sciences, Norwegian University of Life Sciences, P.O. BOX 5003, 1432 Aas, Norway; 3 Department of Crops Genetics, John Innes Centre, Norwich Research Park, Colney Lane, NR4 7UH Norwich, UK

## Abstract

**Background:**

The *def *mutant pea (*Pisum sativum *L) showed non-abscission of seeds from the funicule. Here we present data on seed development and growth pattern and their relationship in predicting this particular trait in wild type and mutant lines as well as the inheritance pattern of the *def *allele in F_2 _and F_3 _populations.

**Findings:**

Pod length and seed fresh weight increase with fruit maturity and this may affect the abscission event in pea seeds. However, the seed position in either the distal and proximal ends of the pod did not show any difference. The growth factors of seed fresh weight (FW), width of funicles (WFN), seed width (SW) and seed height (SH) were highly correlated and their relationships were determined in both wild type and *def *mutant peas. The coefficient of determination *R*^2 ^values for the relationship between WFN and FW, SW and SH and their various interactions were higher for the *def *dwarf type. Stepwise multiple regression analysis showed that variation of WFN was associated with SH and SW. Pearson's chi square analysis revealed that the inheritance and segregation of the *Def *locus in 3:1 ratio was significant in two F_2 _populations. Structural analysis of the F3 population was used to confirm the inheritance status of the *Def *locus in F_2 _heterozygote plants.

**Conclusions:**

This study investigated the inheritance of the presence or absence of the *Def *allele, controlling the presence of an abscission zone (AZ) or an abscission-less zone (ALZ) forming in wild type and mutant lines respectively. The single major gene (*Def*) controlling this phenotype was monogenic and *def *mutants were characterized and controlled by the homozygous recessive *def *allele that showed no palisade layers in the hilum region of the seed coat.

## Background

A *development funiculus *(*def*) mutant pea (*Pisum sativum*) is known as a spontaneous mutation with monogenic recessive inheritance [1-4]. The chromosomal location of the *Def *allele has been found to be located at the bottom end of linkage group VII corresponding to chromosome no 4 [5-7]. Usually in wild type pea, there is a distinctive cell separation between funicle and seed coat that leads abscission of seed and results in detachment of seed from funicle. The wild type pea developed a double palisade layer and these may contribute to seed abscission [8]. In contrast, the palisade layers were absent in the *def *mutant pea and the funicle thus remained firmly attached to the seed coat resulting in non-abscission of seed from the pod. In spite of the distinct phenotypical differences between the wild type and *def *mutant, there was only limited information available on the trait [5,9]. In the present study, we differentiate seed growth and development in the wild type and the *def *mutant lines. The trait inheritance pattern in the *Def *locus was also examined in F_2 _and F_3 _segregate populations by crossing wild type (*Def*) and mutant (*def*).

In pea seeds, growth and development is characterized by three distinct phases and two lag phases [10]. The first phase comprises cell division of cotyledon cells. This phase is also described as pre-storage. The second phase is the maturation step and marks the period where cotyledon cells expand and proteins and starch are laid down as reserve compounds [11]. In the third phase, the maturation process is completed and the seed undergoes a desiccation period. The first lag period in seed growth correspond to a rapid decline in the growth of the testa and disappearance of the endosperm [12,13]. During the second lag phase, the level of the liquid endosperm declines to a minimum and the embryo then makes contact with the internal surface of the testa. The pattern of seed development is important in determining the optimum stage of seed maturation, that produces the maximum quantity and quality of seeds [14]. In plant species, the ovule may develop into a seed non-randomly with respect to ovule position in the ovary and there is a general tendency towards a greater probability of seed maturation at the distal end of the pod [15,16]. Therefore, it was useful to ascertain whether the effect of ovule position may have an effect on the developmental growth pattern in *Def *wild type and *def *mutant pea lines.

Allometric relationships are powerful predictive tools in plant sciences that may be used in predicting a particular a trait from other attributes of the plant [17]. Seed size has been found to be a useful attribute in predicting the ability of plants to establish in drought [18] and nutrient stress [19]. Seed size is an essential component of the life history in plants [20] because any small changes may cause differences in seedling growth, survival, and yield [21]. The funiculus is the only point of attachment between the developing seed and the plant. Nutrients are channelled from the pod via the funiculus to the seed coat [22]. Since the funiculus acts as a conduit through which nutrients pass from the plant into the seed, an allometric relationship between the funiculus and other seed attributes may provide valuable insights into its role in seed development.

In Mendelian genetics, the F_2 _generation of a single monogenetic trait generally produce approximations to a 3:1 ratio depending on population size. However, deviations may occur with regards to the 3:1 ratio described as segregation distortion. Segregation distortion is the deviation of observed genetic ratios from the expected Mendelian ratios of a given genotypic class within a segregating population [23-25]. Lyttle [26], proposed that distorted segregation ratios may be the result from gametophytic competition where there is a preferred choice in fertilization, or the abortion of either the female or male gametes or zygotes. Taylor and Ingvarsson [27], has been specific in attributing the cause of segregation distortion to mechanisms that act in the male gametes. Segregation ratios that do not obey expected Mendelian ratios have been reported in a number of plants including pea (*Pisum sativum*) [28], common bean [29], mungbean (*Vigna radiata *L Wilcek) [30], barley (*Hordeum vulgare*) [31,32], maize (*Zea mays*) [33,34], rice (*Oryza sativa*) [35,36] and wheat (*Triticum aestivum*) [37-39]. Segregation analysis may serve as an important intermediate tool to help investigators plan more sophisticated genomic studies [40] and further enable breeders to manipulate major genes [41,42].

## Results

### Pod length, seed position and seed fresh weight

Pod length was measured to identify differences in growth patterns between *Def *wild type lines (JI 116 and JI 2822) and *def *mutants (JI 1184 and JI 3020) (Tables ^Q4^1 and 2). The pod length in all four accessions increased as the pods matured although there was some variation among accessions. Pod length increased significantly at P3.1 and P5.1 in JI 116 and JI2822, respectively but wild type JI116 showed longer pods than *def *mutant JI 1184. The dwarf type increased significantly at P2.1 in both wild type JI2822 and JI3020 and dwarf mutant JI3020 showed longer pods than JI2822. Through observation and data, we established that pod length increased upon maturity but that pod length was not important factor in the identification of the *def *mutant.

**Figure 1:**
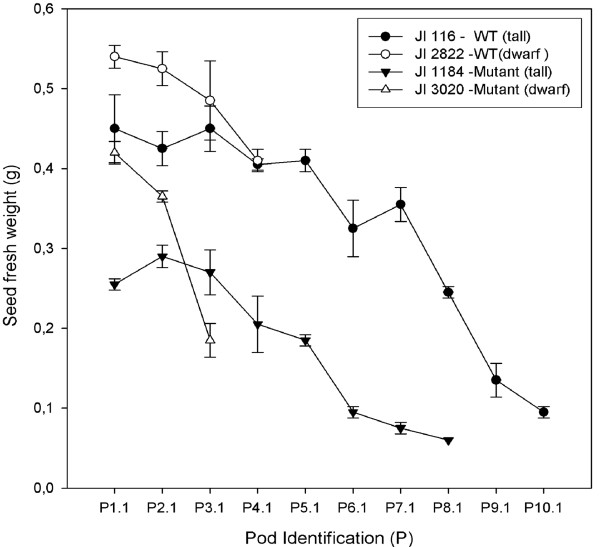
**The relationship between seed fresh weight and pod age in the wild type and the *def *mutant *P. sativum***. JI 116 is tall wild type. JI 2822 is dwarf wild type. JI 1184 is tall mutant and JI 3020 is dwarf mutant. Seeds in the first (most mature) pod and close to pea stock are designated as P1.1. The youngest pod and close to the pea stock is designated as P10.1 for JI 116, P8.1 for JI 1184, P4.1 for JI 2822 and P3.1 for JI 3020.

In order to study whether seed fresh weight was a significant factor in pea seed abscission, we measured seed fresh weight at maximum size in each pod in all the four accessions used in this study. The most mature seed at pod identification P1.1 showed maximum seed fresh weight in all the four accessions whereas the youngest seeds (P10.1 in JI 116, P8.1 in JI 1184, P4.1 in JI 2822 and P3.1 in JI 3020) showed minimum seed fresh weight (Figure 1). Among accessions, seeds of dwarf types (JI 2822 and JI3020) were heavier and had slightly larger hilum areas than the tall types (JI 116 and JI1184), respectively (data not shown). The wild type pea seeds (JI 116 and JI 2822) were heavier than *def *mutant types (JI 3020 and JI 1184) seeds indicating that seed fresh weight may be an important factor in maturity and abscission event.

To study whether seed position has an effect on seed fresh weight, the seed fresh weight at the proximal and distal positions in the pod were measured. We observed a steady increase in seed fresh weight in both the proximal and distal locations in the pods of all accessions as they matured (Additional file 1: Table 1). In both proximal and distal location of seeds in JI 116 (wild type, tall), seed fresh weight increased in a similar way and there was no significant differences in seed fresh weight between proximal and distal locations. In addition, the interaction between proximal and distal seed locations was not significant in JI 1184, JI 2822 and JI 3020. Thus we established that the seed position did not have an effect on seed fresh weight.

**Table 1:** Details of *Pisum sativum *accessions and their allelic status with respect to the *Def *locus

Accession	Name	*Def *allele
JI 116	cv. Parvus	*Def *(wild type)

JI 2822	RIL, research line	*Def *(wild type)

JI 1184	Priekuskij-341-*def*	*def *(mutant)

JI 3020	cv. Nord	*def *(mutant)

### Seed growth and development

**Figure 2:**
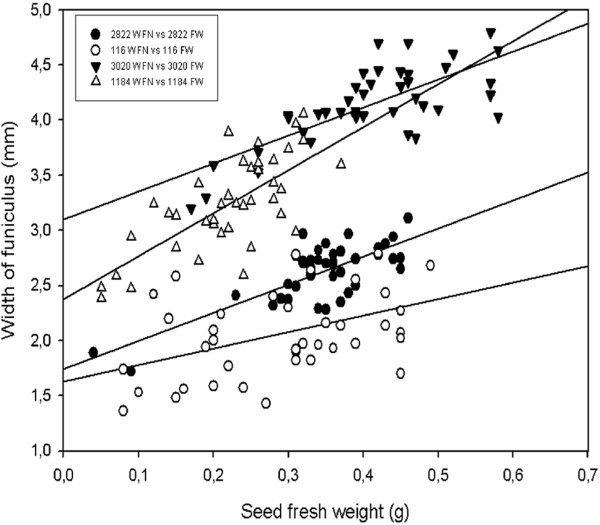
**The relationship between seed fresh weight (FW) and width of funiculus (WFN) in the wild type and the *def *mutant *Pisum sativum***. Linear models were fitted to four data sets: JI 116 (*Y *= 1.63 + 1.49 *X*; *R*^2 ^= 19.6%; *P *= 0.000), JI 2822 (*Y *= 1.74 + 2.55 *X*; *R*^2 ^= 57.4%; *P *= 0.000), JI 1184 (*Y *= 2.37 + 3.91 *X*; *R*^2 ^= 51.7%; *P *= 0.000), JI 3020 (*Y *= 3.10 + 2.54 *X*; *R^2 ^*= 55.9%; *P *= 0.000).

**Figure 3:**
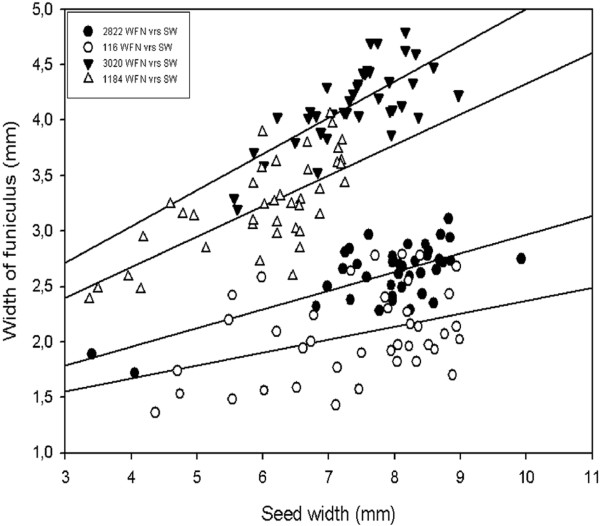
**The relationship between seed width (SW) and width of funiculus (WFN) in the wild type and the *def *mutant *Pisum sativum***. Linear models were fitted to four data sets: JI 116 (*Y *= 1.20 + 0.117 *X*; *R*^2 ^= 14.8%; *P *= 0.001), JI 2822 (*Y *= 1.28 + 0.169 *X*; *R*^2 ^= 49.0%; *P *= 0.000), JI 1184 (*Y *= 1.57 + 0.276 *X*; *R*^2 ^= 48.2%; *P *= 0.000), JI 3020 (*Y *= 1.74 + 0.326 *X*; *R*^2 ^= 53.2%; *P *= 0.000).

**Figure 4:**
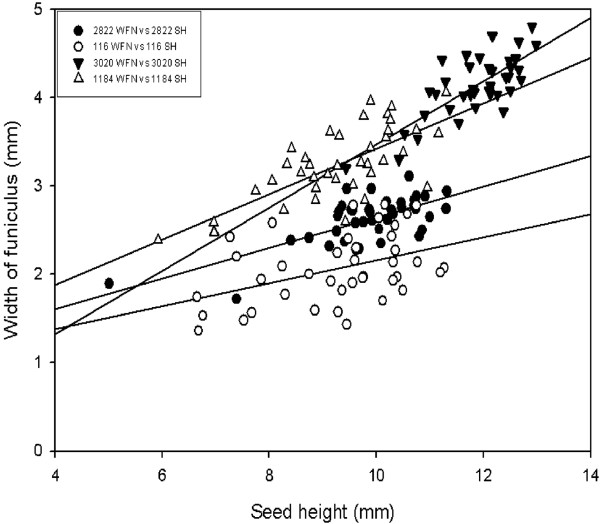
**The relationship between seed height (SH) and width of funiculus (WFN) in the wild type and the *def *mutant *Pisum sativum***. Linear models were fitted to four data sets: JI 116 (*Y *= 0.855 + 0.131 *X*; *R*^2 ^= 17.9%; *P *= 0.051), JI 2822 (*Y *= 0.909 + 0.173 *X*; *R*^2 ^= 49.5%; *P *= 0.003), JI 1184 (*Y *= 0.851 + 0.257 *X*; *R*^2 ^= 53.5%; *P *= 0.024), JI 3020 (*Y *= -0.114 + 0.359 *X*; *R^2 ^*= 56.6%; *P *= 0.852).

For further growth and development analysis, the width of the funiculus (WFN), seed fresh weight (FW), seed width (SW), seed height (SH) and their relationships were studied. A linear relationship between the WFN and the FW was observed in all four accessions (Figure 2). The best relationship was observed in wild type JI 2822. The relationship between the WFN and the FW was described using the model WFN = 1.74 + 2.55FW which explained 57% of the variation in WFN. The relationship between the WFN and SW was best explained in the mutant JI 3020 using the model WFN = 1.74 + 0.326 SW accounting for 53.2% of the variation (Figure 3). A lower correlation was observed in JI 116 with an *R^2 ^*value of 14.8%. In addition, the best relationship between WFN and SH was found in mutant JI 3020 using the model WFN = -0.114 + 0.359SH (Figure 4). *R*^2 ^values for all the models describing the relationship between WFN and other predictors are presented in additional file 2: Table 2.

**Table 2:** Changes in the pod length of wild type (JI 116 and JI 2822) and *def *mutant (JI 1184 and JI 3020) peas (*Pisum sativum*) at various developmental growth stages

Growth stage	Mean cultivar pod length (mm)			
	
	JI 116	JI 2822	JI 1184	JI 3020
P8.1	55.7 ± 4.6b		34.0 ± 9.4a	

P7.1	59.3 ± 4.7ab		40.3 ± 7.7a	

P6.1	61.0 ± 2.1 ab		49.3 ± 3.5a	

P5.1	64.73 ± 2.6ab		52.0 ± 1.2ab	

P4.1	65.3 ± 2.7ab	46.7 ± 3.3b	58.0 ± 1.2ab	

P3.1	68.3 ± .5a	53.0 ± 3.6ab	57.0 ± 1.5ab	59.7 ± 4.4a

P2.1	67.3 ± 1.3a	55.3 ± 2.3a	56.3 ± 1.8ab	67.7 ± 3.4b

P1.1	67.7 ± 1.4a	55.3 ± 2.7a	59.7 ± 0.35b	70.7 ± 2.9b

Mean ± SE followed by different letters in the same column is significantly different at *P *= 0.05 by the Tukey simultaneous comparison

When FW together with SW were included in the model, wild type JI 2822 with *R^2 ^*59.2% gave the best predictive ability with the regression equation WFN = 1.52 + 1.89FW + 0.0572SW. A model with FW and SH included in the regression of JI 3020 gave an additional variance in the WFN term with a *R^2 ^*value of 62.7% and the regression equation WFN = 1.15 + 1.39FW + 0.204SH. Again, JI 3020 gave the best predictive value when SW and SH were included in the model resulting in the regression equation WFN = 0.113 + 0.181SW + 0.227SH. WFN had an improved linear relationship when FW, SW and SH were all included in the model. In all the four accessions tested, *def *cultivars JI 3020 and JI 1184 with a *R^2 ^*value 65.3% and 55.1% respectively, explained much of the variation in WFN as compared to *Def *accession JI 2822 and JI 116 with *R*^2 ^values of 59.2% and 21.7% respectively. The regression equation explaining much of the variation in JI 3020 was WFN = 0.155 + 0.052FW + 0.176SW + 0.225SH, *R^2 ^*= 65.3%. We included interaction terms, FW*SH, FW*SW and SW*SH in the model which comprised all the three predictors described. Additional variation was observed in the dwarf *def *accession JI 3020 when the interaction terms FW*SH, FW*SW and SW*SH were included giving predictive *R^2 ^*values of 66.3%, 68.7% and 66.6% respectively. We also looked at the inclusion of the interaction term FW*SH*SW to explain variation in WFN. We recorded a predictive value of 67.7% to explain the variation in the hilum size of the *def *JI 3020 as a result of the inclusion of an interaction term which included all the predictors in these study and these values were higher than those recorded for JI 2822, JI1184 and JI 116. The backward elimination method over pooled data from all four accessions was used to select and validate a model that best explained the relationship between WFN and the predictors as well as their interaction terms. Our results showed that variation of WFN of pea seeds was best associated with SH and SW (*R*^2^, 70.40; PRESS, 35, 6104).

**Figure 5:**
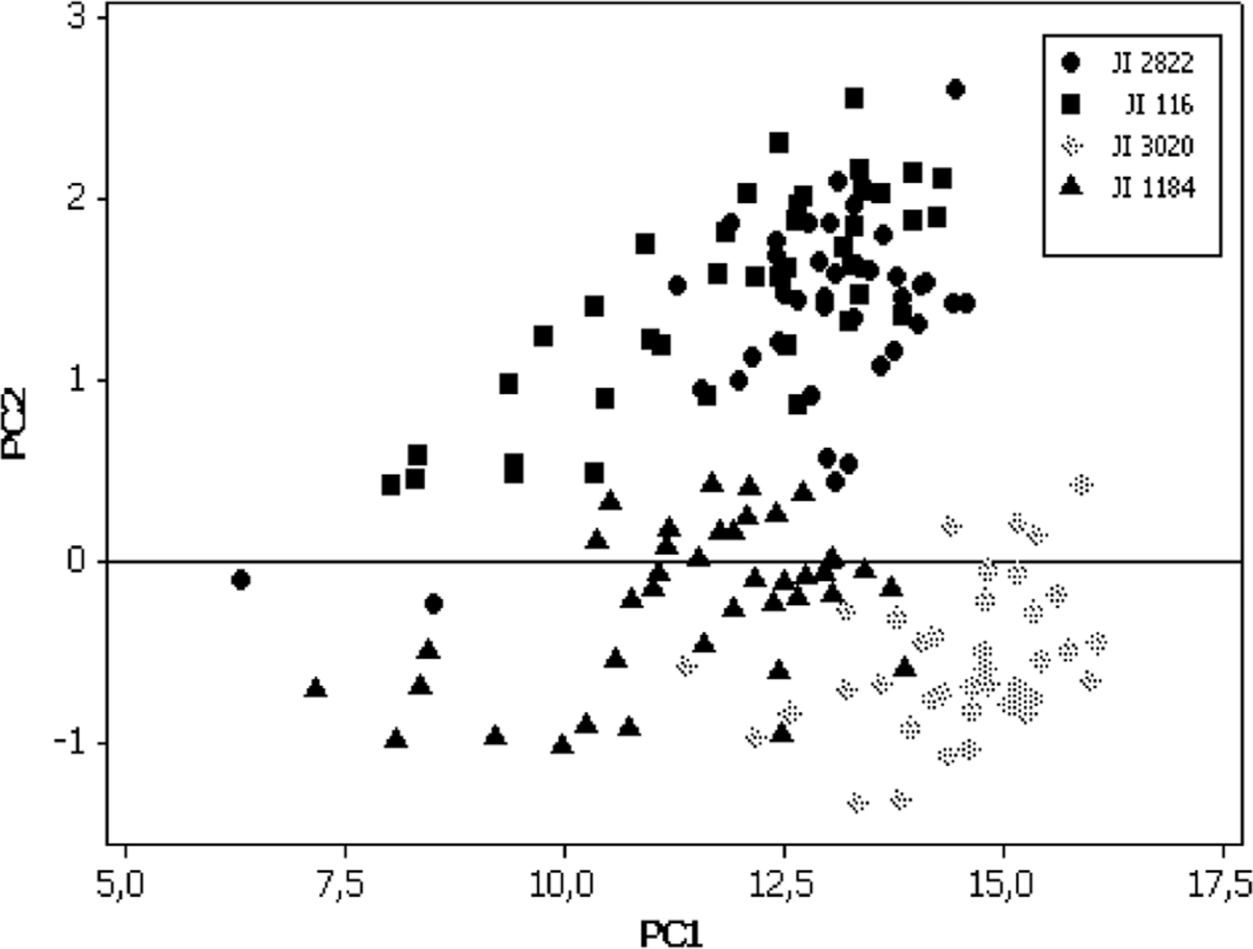
**Principal component analysis (PCA) of the overall growth variation among two *Def *wild types and two *def *mutant peas**.

Principal component analysis (PCA) applied to overall growth variation among two *Def *wild types and two *def *mutant types showed a 96% discrimination could be obtained using only two PCs (Figure 5). We observed differences among all four accessions. The PC1 grouped mutant with *def *loci, tall JI 1184 and dwarf JI 3020 separately from wild types JI 116 and JI 2822. In PC2, clusters were formed between tall (JI 116 and JI 1184) and dwarf phenotype (JI 2822 and JI 3020), thus grouping them together (Figure 5).

### Segregation analysis in two F_2 _populations

F_1 _plants produced from crosses JI 2822 × JI 1184 and JI 2822 × JI 3020 were selfed to produce two different populations of F_2_. In population one (JI 2822 × JI 1184), we observed the segregation pattern of the *Def *locus in two separate Mendelian ratios. When the ratio 3:1 was tested in population one, homozygous *Def*/*Def *seeds could not be distinguished phenotypically from heterozygous *Def*/*def *seeds and chi square (*χ*^2^) analysis gave a value of 0.1212 (*P*, 0.05) (Table 3) hence we failed to reject the null hypothesis. When the ratio 1:2:1 was considered, F_2 _progeny, produced by crossing *Def*/*def *heterozygotes segregated into homozygous *Def*/*Def*, heterozygous *Def*/*def *and the homozygous *def*/*def *classes. However, *χ*^2 ^value of 25.590 reveal that the observed ratios differ significantly from the expected ratios and therefore we rejected the null hypothesis. In the second population (JI 2822 × JI 3020), F_2 _plants were produced from short F_1 _heterozygous (phenotypically similar to *Def*/*Def*) plants (Table 4). The Mendelian ratios 3:1 revealed in chi square analysis that the F_2 _progeny also segregated into two main classes, homozygous *Def*/*Def *and another homozygous line *def*/*def*. The *χ*^2 ^values from the 3:1 test were 0.0666. However when the ratio 1:2:1 was tested, three genotypic classes were segregated in the F_2 _seeds *Def*/*Def*: *Def*/*def*: *def*/*def *(1: 2: 1). The *χ*^2 ^values were 56.1 and could reject the hypothesis. Therefore we accepted the null hypothesis in the 3:1 ratio.

**Table 3:** F_2 _plants from selfing of the F_1 _progeny resulting from the cross JI 2822 × JI 1184

	Genotype	Observed number (O)	Expected number (E)	Difference (O-E)	(O-E)^2^/E
*3: 1*	*Def*/*Def*	34	33	1	0.03030
	
	*Def*/*def*	-	-	-	-
	
	*def*/*def*	10	11	--1	0.09090
	
	Total	44	44	0	0.1212 = χ_calc_^2^

*1: 2: 1*	*Def*/*Def*	25	11	14	17.681
	
	*Def*/*def*	9	22	--13	7.681
	
	*def*/*def*	10	11	--1	0.0919
	
	Total	44	44	0	25.590 = χ_calc_^2^

* 3:1 Observed values do not differ significantly from expected values at 1 degree of freedom and 0.05 levels of significance

*1: 2: 1 Observed values differ significantly from the expected value at 1 degree of freedom and 0.05 level of significance

**Table 4:** F_2 _plants from selfing of the F_1 _progeny resulting from the cross JI 2822 × JI 3020

	Genotype	Observed number (O)	Expected number (E)	Difference (O-E)	(O-E)^2^/E
*3: 1*	*Def*/*Def*	59	60	--1	0.01666
	
	*Def*/*def*	-	-	-	-
	
	*def*/*def*	21	20	1	0.05
	
	Total	80	80	0	0.0666 = χ_calc_^2^

*1: 2: 1*	*Def*/*Def*	47	20	27	36.45
	
	*Def*/*def*	12	40	--28	19.60
	
	*def*/*def*	21	20	1	0.05
	
	Total	80	80	0	56.1 = χ_calc_^2^

* 3:1 Observed values do not differ significantly from expected value at 1 degree of freedom and 0.05 level of significance

* 1: 2: 1 Observed values differ significantly from the expected value at 1 degree of freedom and 0.05 level of significance

### Histological and developmental analysis of F_3 _segregants

**Figure 6:**
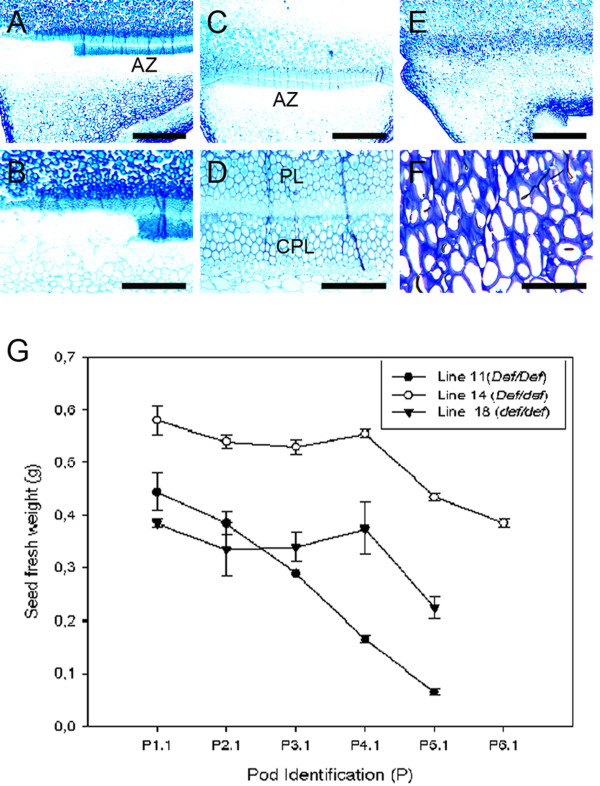
**Light micrographs and seeds growth of F3 populations resulting from crosses between tall mutant type (JI 1184) by dwarf wild type (JI 2822)**. (**a-f**) Light micrographs of segregant F3 sections. (**a-b**) Heterozygote genotype *Def*/*def *(+-) line 14. **a **Abnormal and partial palisade layer. **b **Higher magnification of A. (**c-d**) Homozygous dominant genotype *Def*/*Def *(++) line 11. **c **Appearance of the normal AZ palisade layer that reveals the typical double palisade layer. **d **Higher magnification of C. (**e-f**) Homozygous recessive genotype *def*/*def *line 18. **e **There is no visible palisade layer. **f **Higher magnification of E. **g **Relationship between seed fresh weight and pod age in F3 plants. *AZ *Abscission zone, *CPL *Counterpalisade layer, *PL *Palisade layer. *Bars *= 12.5 μm (a, **c**, e); 25 μm (**b**, **d**, **f**).

In order to confirm that the *Def *locus is monogenic, we crossed JI 2822 and JI 1184 which were then selfed to obtain the F2. The F2 generation was then grown to obtain seeds from the F_3_. We selected three lines from the F_3 _population and they included heterozygote line 14 (*Def*/*def*), a dominant homozygous line 11 (*Def*/*Def*) and a recessive homozygous line 18 (*def*/*def*). Observations of histological data confirmed the 3:1 ratio (data not shown). However, we found an interesting aspect in the heterozygote line 14 that exhibited a partially formed palisade layer (Figure 6a). This partial palisade layer was observed in line 14 from 9 heterozygous lines from a total of 44 lines. Higher magnification of the partially formed palisade layer revealed the apparent absence of the counter palisade layer and yet the abscission process occurred nevertheless in the area where there is the partial counter palisade layer (Figure 6b). A homozygous dominant line 11 showed distinct double palisade layers and abscission development (Figure 6c and 6d) while a recessive (line 18) showed the lack of a palisade layer resulting in a non-abscission process (Figure 6e and 6f). The observed numbers of F_3 _pod identification of the selected F_3 _segregants was an average of numbers of pod identification in parental JI 2822 and JI 1184. All the three lines showed a correlation between pod maturity and seed fresh weight with seed fresh weight generally increased upon pod maturity (Figure 6g).

**Figure 7:**
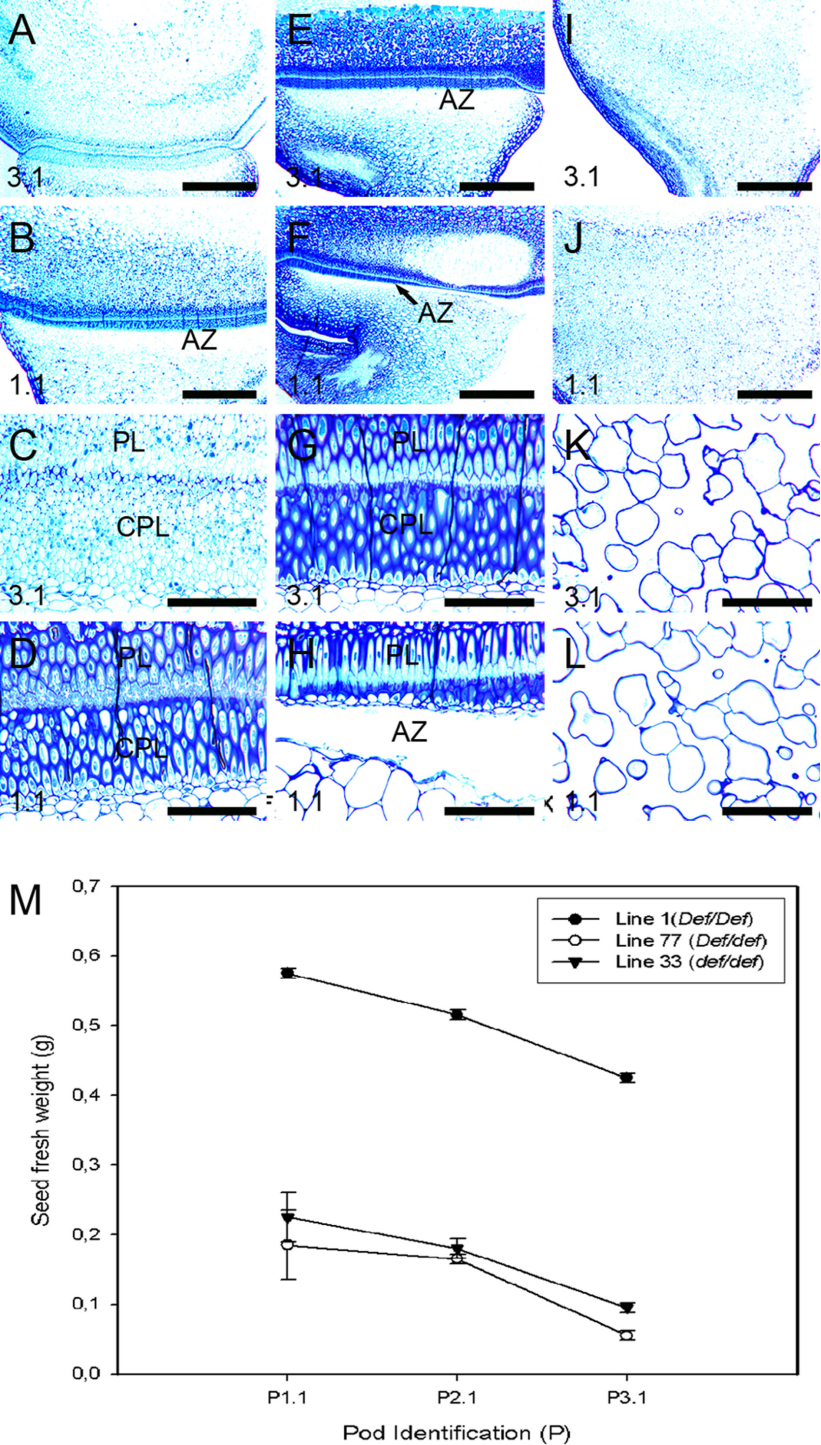
**Light micrographs and seeds growth of F3 populations resulting from crosses between dwarf wild type (JI 2822) and dwarf mutant pea (JI 3020)**. (**a-l**) Light micrographs of segregant F3 sections. (**a-d**) Heterozygote genotype *Def*/*def *(+-) line 77. **a **Young seed of line 77 shows a less differentiated palisade layer. **b **Mature seed section of line 77 shows normal double palisade layer development. **c-d **Higher magnification of (**a**) and (**b**), respectively. (**e-h**) Homozygous dominant genotype *Def*/*Def *(++) line 1. **e **Young seed section of line 1. **f **Mature seed section of line 1. **g-h **Higher magnification of (**e**) and (**f**), respectively. **i-l **Homozygous recessive genotype line 33. (**i**) Young seed section of line 33 shows no distinct palisade layer. (**j**) Mature seed section of line 33. (**k**)**-**(**l**) Higher magnification of (**i**) and (**j**), respectively. (**m**) Relationship between seed fresh weight and pod age in F3 plants. *AZ *Abscission zone, *CPL *Counterpalisade layer, *PL *Palisade layer. *Bars *= 12.5 μm (**a**, **b**, **e**, **f**, **i**, and **j**); 25 μm (**c**, **d**, **g**, **h**, **k**, and **l**).

We further analyzed the significance of the 3:1 ratio through structural examination of the second F_3 _population produced from a cross between JI 2822 (dwarf wild type) and JI 3020 (dwarf mutant). Structural examinations of selected F_3 _segregants from this cross (heterozygous line 77, homozygous dominant line 1 and homozygous recessive line 33) were similar to parental. The double palisade layer was seen in heterozygous line 77 and the homozygous dominant line 1 (Figure 7a-d). However, the palisade layer in the young seed (P 3.1) of the heterozygous line 77, was less differentiated (Figure 7a and 7c) compared to the fully differentiated palisade layer in the young seed of homozygous dominant line 1 (Figure 7e and 7h). At higher magnification, the distinct separation of the palisade layers in the young heterozygous line 77 was less conspicuous (Figure 7c) compared to the young seed in the homozygous dominant line (Figure 7g). In the homozygous recessive line 33, the abscission process was not observed (Figure 7i and 7j) and we observed that cells of a parenchymatous nature were highly irregular in shape (Figure 7k and 7l). The AZ in the homozygous dominant line 1 was clearly defined with the cell separation event at an advance stage (Figure 7f and 7h). The seed fresh weight at pod identification P3.1 (younger stage) in heterozygous line 77 and homozygous recessive line 33 were lighter compared to homozygous dominant line 1 (Figure 7m). Similarly, seed fresh weight at pod identification P1.1 (mature stage) in homozygous dominant line 1 was heavier than the heterozygous line 77 and homozygous recessive line 33 and the homozygous dominant line 1 showed a more cell to cell separation in the AZ (Figure 7h).

## Discussion

### Abscission of seeds in wild type and *def *mutant pea

The abscission of pea seeds can be defined as seed separation from the funicle [43]. This study focused on growth and development of seeds in two wild type lines and two *def *mutants lines. Sometimes abscission can be considered as the last step of organ developments and may be accompanied by senescence or aging [44]. The abscission process in wild type peas is correlated to seeds maturity since mature seeds easily abscised while young seeds fail to show any discernible abscission event. In previous work [8], a structural comparison between wild type pea and the *def *mutant showed different structural phenotype. The wild type underwent a normal abscission between the funicle and seed coat at maturity while *def *mutant exhibited a non-abscission event in the seeds. Therefore, the abscission event is highly related to seed maturity and the *def *locus is important in controlling the abscission event in pea seeds.

Our results showed that pod length increased and finally stabilized in mature pods. In all accessions, the initial increase in pod length is generally associated with initial seed filling process [45]. It is interesting to note that with the exception of tall mutant (JI 1184) the initial significant increase in pod length occurred at growth stage P7.1 whilst the in the tall mutant line it occurred at P5.1. This is not surprising since the time to anthesis was much longer in the tall JI 1184 than in the other accessions.

Seed fresh weight was measured to study the developmental growth pattern in seeds. In general, seed development in pea consisted of two growth phases and separated by two lag phases [10,46]. However, Carr and Skene [47], revealed a biphasic growth pattern separated by a single lag phase in French beans (*Phaseolus vulgaris *L). In our results obtained in the proximal and distal location of seeds in JI 116, an initial increase in seed fresh weight was recorded from P8.1 to P7.1 in the proximal end of pod whereas in the distal end, significant growth occurred from P8.1 to P6.1 (Table 2). The same trend was observed in JI 1184 at both proximal and distal ends of the pod. In both dwarf wild type and *def *mutant types, initial growth rates were significant in both proximal and distal locations except for the distal part of JI 2822. The initial growth phase in all the accessions is due to cell division and associated with changes in the embryo, seed testa and endosperm [10]. Even though we observed a steady increase in seed fresh weight at the proximal and distal ends in tall wild type JI 116, rather slow growth was seen between P4.1 and P3.1 at the proximal end. At the distal end of JI 116, seed fresh weight changes were significant between developmental growth stage P4.1 and P2.1. This period of growth may represent overlaps in transition from the second growth phase to maturation phase. In the tall *def *mutant JI 1184, a steady increase after the initial seed growth was observed at both the proximal and distal locations of seeds in the pod until maximum seed fresh weight was reached at P2.1 at both proximal and distal seed locations. This steady increase in seed fresh weight until the maximum seed fresh weight was attained may represent a steady transition and attainment of seed maturation phase. In dwarf wild type JI 2822, maximum seed fresh was obtained at P2.1 at the proximal end and decreased at P1.1 indicating a maturation phase. However, maximum seed fresh weight at the distal end was 0.5 g and we found no lag phase. In dwarf *def *mutant type the growth pattern appear linear at both proximal positions. This linear accumulation of seed fresh weight in JI 3020 may suggest that there was the possibility of delayed termination of reserve accumulation as suggested by Chinnasamy and Bal [14] in grass pea seeds. At both proximal and distal ends, there was no appreciable increase in seed weight at P1.1 indicating the onset of the desiccation period where the seeds begin to dry. Generally, in both tall wild types and mutant types, there was no pronounced lag phase. This may be due to the choice of the developmental growth stages used in this study that did not cover this event. Seed maturation is an important phase in seed development and marks the termination of the growth of the embryo [48] prior to seed desiccation phase where the seed loses water and passes into a dormant stage [49]. In the present study seed maturation was observed in all lines to have occurred at P1.1. However, we observed that maturation occurred at the proximal end in JI 2822 at seed developmental stage (P2.1) followed by a well defined desiccation period at P1.1 where there was seed weight loss.

Generally there was a positive correlation between repressors (WFN) and all the predictors described in this study. Our results showed a better predictive value (*R^2^*, 57.4%) and a positive correlation between WFN and FW in dwarf wild type (JI 2822) than tall wild type and mutant types. The observed relationship between WFN and FW obtained was consistent with the findings of Mawson et al. [50] who found that the size of the funiculus increases from an early developmental stage to a more advanced stage. WFN correlated positively with SW except for JI 116 (*R^2^*, 14.8%). The same pattern was observed when SH was regressed on WFN in *def *mutant lines than *Def *wild type lines. The general trend observed where mutants types exhibited better *R^2 ^*values than wild types (Additional file 2: Table 2) may be due to the fact that mutants, particularly JI 3020 (dwarf, mutant) have a much more swollen funiculus than in wild types (data not shown). A combination of two of the predictors as well as all three predictors and their interaction terms were included in the fitted models. The best predictive value (*R^2^*, 68.7%) was recorded in dwarf JI 3020 when the interaction term FW*SW was included in the fitted model as compared to where the interaction term FW*SW*SH was included. Thus it may be suggested there could be a relationship involving fresh weight and seed width. However, when data were pooled together and backward elimination of terms applied, the model excluded seed FW but included SH and SW in determining the best relationship between predictors and WFN. The results obtained from seed development and regression analysis involving all the four accession revealed differences. We further used PCA analysis to confirm and summarized the differences between the accessions.

Segregation distortion may be explained as a deviation from expected ratios in a given phenotype or genotypic progenies within a segregating population [33]. In this work, we have used Pearson's chi square analysis to test and explain phenotypic ratios and quantify deviations in 3:1 and 1:2:1 Mendelian ratio when *Def*/*Def *wild type was crossed to *def*/*def *mutant. In the F_1 _progeny produced in the initial crosses, heterozygous *Def*/*def *obtained were not phenotypically different from the homozygous dominant (*Def*/*Def*) parents. Our results showed consistency with what would have been expected if the abscission event in pea wild type lines are under the control of a single dominant gene, *Def *[51]. Phenotypic segregation of F_2 _seeds showed that observed numbers obtained from a 1:2:1 ratio in the two populations tested was insignificant as revealed by *χ*^2 ^values (25.590 and 56.1). These results agree with the work of Kloos et al. [52], who showed using chi square analysis that the inheritance of dark disk colour was determined by a single dominant gene. Chi square analysis have also been used to investigate excess or deficiency segregation distortion in F_2 _populations of pea recombinant inbred populations [53]. Genetic analysis based on chi square analysis in two self pollinating pea plants revealed that embryo abortion was the cause of segregation distortion when the ratio 9:3:1 was tested [28]. Even though we did not observe large deviations from our 3:1 ratio, some of the factors that may be responsible for the deviations in the 1:2:1 ratios in the two populations, may include the presence of lethal genes that may be involved in the various stages of reproduction including sporogenesis, fertilization and seed development [54,55].

### Structural analysis of F_3 _segregants

We used the dwarf wild type JI 2822 in crosses with tall mutant types JI 1184 and dwarf mutant JI 3020 because JI 2822 has a black hilum surface which can be readily observed during the maturation phase. Black hilum is a good segregating morphological marker offering a clear visual reference to tissue differentiation at the macro level. The results of our genetic inheritance studies through cell structure analysis suggest that *Def *is determined by a one-locus diallelic system, indicating a single gene hypothesis. Structural analysis involving the dwarf wild type (JI 2822) and tall mutant (JI 1184) which were characterized genotypically as homozygous dominant lines (*Def*/*Def*), heterozygous lines (*Def*/*def*) and homozygous recessive lines (*def*/*def*). Phenotypic observation through structural analysis revealed that the heterozygous lines were similar to the homozygous dominant lines (data not shown). However, an aberrant or incomplete phenotype showing partial appearance of the palisade layer was observed in a heterozygous line from this cross (Figure 5a and 5d). A possible explanation for this aberrant phenotype may be that it arose through a spontaneous event as described for the inheritance of *homostyles *by Tamari [56]. Such abnormality may be attributed to a number of reasons including physiological, molecular and environmental factors [36]. The second F_3 _population (JI 2822 × JI 3020) confirmed the 3:1 segregation ratio in pea and no aberrant phenotypes among the heterozygous progeny was observed. The less differentiated palisade layer observed in the young seed of the heterozygous line 77 (Figure 6a), may possibly be due to the early developmental stage (Figure 6m). This is because the epidermal cells at that stage were made of cuboidal epidermal cells before the fully differentiated epidermal cells appear at stage P1.1 with a mature seed (Figure 6d) as described by Miller [57]. Generally, seed weight in F_3 _lines correlated with the formation of an AZ. Mature seeds had a clearer AZ than young seeds in *Def *wild type. Generally, seed weight correlated with the maturity of the pod on the pea plant. The similar size of the F_3 _progenies to that of parental may suggest the influence of a maternal effect in seed maturation in the F_3 _progenies due to access to resources from the maternal seed [58]. The interactive influence of maternal photoperiod and temperature has also been hypothesized to control the molecular expression in progenies of Norway spruce [59]. However, it has also been suggested that final seed weight may be determined by genetic, environment and frequently significant genotype × environment interactions [60].

## Conclusions

The palisade and counter-palisade layers, by default is the structure that delimits the seed coat and the funicle in the wild type pea carrying the *Def *locus and is thus the location for where the abscission of the funicle from the seed occurs. This layer was conspicuously absent in the mutant cultivars that carry the recessive *def *allele. In addition, we confirm earlier findings through anatomical analysis that the inheritance of the AZ defined by *Def *allele was controlled by the *Def *locus [51]. We observed the presence of a partially formed palisade layer and a less differentiated palisade layers in young seeds in two F_3 _populations. However, the number of seeds with such phenotypes were small and insignificant compared to the significant 3:1 segregation ratio. This study investigated the inheritance of the presence or absence of the *Def *allele, controlling phenotypes where there is AZ or an ALZ formation in wild type and mutant lines respectively. The single major gene (*Def*) controlling this phenotype was identified by Mendelian genetic analysis, confirming earlier findings on the *def *allele [51].

## Methods

### Plant materials

The four lines of pea (*Pisum sativum *L.) seeds JI 116, JI 2822, JI 1184 and JI 3020 used in this study were selected on the basis of the presence of specific alleles at the *Def *locus, which control the detachment of the seed from the funicle (Table 1). JI 1184 originates from Rozenthal's collection from Russia where the *def *mutation was first identified and isolated and is an early line selected as carrying the *def *allele. It has been used for agronomic studies and is a sister line to the type line for *def *mutant allele. JI 3020 is a registered cultivar from the Netherlands that incorporates the same mutant *def *allele. In the absence of near-isogenic lines for the *Def *alleles, two well characterized lines (JI 116 and JI 2822) that matched the gross plant habit of the mutant lines were selected. Both these lines are well characterized genetically and were selected for use in genetic analysis of heterozygous *Def*/*def *seeds that are the subject of further study of this locus.

Seeds corresponding to each line were sown in pots with fertilised peat (Floralux, Nittedal Torvindustrier, Norway) and grown under greenhouse conditions at 22°C and 16/8 h photoperiod with a photon flux of 110 μmol m^-2 ^s^-1 ^(400-700 nm Photosynthetic Active Radiation (PAR)) and a day length extending light provided from incandescent lamps (OSRAM, Germany).

Seeds from two populations were produced from crosses JI 3020 × JI 2822 (population one) and JI 1184 × JI 2822 (population two). The F1 from the two populations were self-crossed to produce F_2 _plants.

### Definition of pod identification and measurement of growth

The development of the abscission process was shown to correlate with maturity of seeds, by measuring seed fresh weight at each pod identification stage. Seeds in the first (most mature) pod and close to pea stock are designated as P1.1. The youngest pod and close to the pea stock is designated as P10.1 for JI 116, P8.1 for JI 1184, P4.1 for JI 2822 and P3.1 for JI 3020. This system was also applied to seeds in F_3 _populations. Seed fresh weight at corresponding pod identification stages were measured in F_3 _populations

Pod length, proximal and distal seed fresh weights corresponding to each developmental growth stage of the four accessions (JI 116, JI 1184, JI 2822 and JI 3020) were measured. Forty seeds per accession were harvested after maturity in all the four accession. Seeds were randomly selected through the entire developmental series. The width of the funiculus on the seed (WFN), fresh weights (FW), seed width (SW) and seed height (SH) were measured and then analysed statistically.

### Statistical analysis

Means and standard deviations of pod length and seed fresh weight were computed using the Tukey simultaneous comparison test (Minitab version 15). The relationships between WFN and FW, SW and SH were evaluated by fitting regression models with Multiple Linear Regression (MLR) analysis procedure of Minitab (Version 15). The internal validity of the models was tested by coefficient of determination (*R*^2^). Model validations of a pooled data of all the accessions were carried out using the stepwise elimination option (Minitab version 15). We also used Principal Components Analysis (PCA) [61], to find clusters between pea *Def *wild type and *def *mutant lines. In addition, pearson's goodness of fit test was used to study two segregations ratios (3:1 and 1:2:1) to interpret phenotypic ratios and quantify the various deviations expected by chance as described by Halliburton [62].

### F_3 _plant material

Segregation patterns in the inheritance of the *Def *locus were studied in two F_3 _populations produced by crossing (Table 5). Selected seeds lines from two F_3 _populations were produced from crosses between parental JI 2822 × JI 1184 (population one) and JI 2822 × JI 3020 (population two). The F_1 _from the two populations were selfed to produce F_2 _plants. F_2 _populations were grown under same conditions described above to produce F_3 _seeds in two populations and then used for structural examination and seed growth measurement.

**Table 5:** Genetic characterization of two populations of F3 lines used for structural analysis

Cross	Phenotypic description	F3 population (Line)	Presence/absence of *Def *locus in F3 lines
JI 2822 × JI	Dwarf wild type × Tall mutant	1	+ +
		
		77	+-
		
		33	- -

JI 2822 × JI 3020	Dwarf wild type × Dwarf mutant	11	+ +
		
		14	+ -
		
		18	- -

+ + Homozygous dominant (*Def*/*Def*); + -- Heterozygous dominant (*Def*/*def*); -- -- Homozygous recessive (*def*/*def*)

### Plant tissue preparation and examination

For structural analysis, seeds of all lines were embedded in LR White resin (London Resin Company, England) as mentioned in Ayeh et al. [8] Briefly, seeds were cut into 2 mm thick and immediately fixed in 1% formaldehyde, 0.025% glutaraldehyde, 0.1% (*v*/*v*) Tween 20 in 0.01 M sodium phosphate buffer, pH 7.2 and vacuum infiltrated for 1 h. The fixed tissues were placed at 4°C overnight and then dehydrated in a graded ethanol series. After infiltration, the specimens were embedded in LR White and polymerised at 50°C for 24 h. For histological staining, sectioned materials (1 μm thick) were stained with toluidine blue O (Sigma, USA) and mounted in Depex (BDH, USA). Sections were examined using a Leica brightfield microscope (Leica, Germany).

## Competing interests

The authors declare that they have no competing interests.

## Authors' contributions

KOA contributed to the growing of the plants, harvested materials, carried out the experimental examination and drafted the manuscript. YKL participated in designing the experiments, structural analysis and the drafting of the manuscript. MA contributed with plant material, the general idea of the study and participated in revision of the manuscript. AKHE participated in the general idea of the study, the design of the experiments and contributed to the writing and revision of the paper. All authors have read and approved the final manuscript.

## Supplementary Material

Additional file 1 Click here for file

Additional file 2 Click here for file
