# Supplementary material for: Growth, seed development and genetic analysis in wild type and Def mutant of Pisum sativum L
Source: BMC Res Notes. 2011 Nov 11;4:489. doi: 10.1186/1756-0500-4-489 (PMC3231984; doi:10.1186/1756-0500-4-489)
Supplement: Additional file 1 [file 1756-0500-4-489-S1.DOC]

**Additional File 1: Table 1.** Changes in the seed weight at the proximal and distal positions in pods of wild type (JI 116 and JI 2822) and def mutant (JI 1184 and JI 3020) peas (*Pisum sativum*) at various developmental growth stages.

| Growth  Stage | Mean cultivar fresh weight (g) | | | | | | | |
| --- | --- | --- | --- | --- | --- | --- | --- | --- |
|  | JI 116 | | JI 2822 | | JI 1184 | | JI 3020 | |
|  | Proximal | Distal | Proximal | Distal | Proximal | Distal | Proximal | Distal |
| P8.1 | 0.12± 0.06a | 0.13±0.06a |  |  | 0.04± 0.01a | 0.11±0.05a |  |  |
| P7.1 | 0.23± 0.15ab | 0.22±0.08ab |  |  | 0.05± 0.02ab | 0.08±0.01ab |  |  |
| P6.1 | 0.27± 0.07bc | 0.23±0.06abc |  |  | 0.09± 0.03abc | 0.10±0.02abc |  |  |
| P5.1 | 0.32± 0.06bcd | 0.30±0.07bcd |  |  | 0.13± 0.02cd | 0.14±0.02bcd |  |  |
| P4.1 | 0.36± 0.06cde | 0.37±0.06bcde | 0.36± 0.03a | 0.31±0.10a | 0.21± 0.02de | 0.19±0.03de |  |  |
| P3.1 | 0.37± 0.05def | 0.38±0.06 def | 0.43±0.03ac | 0.42±0.03a | 0.26± 0.02ef | 0.25 ± 0.02de | 0.19± 0.12a | 0.19±0.12a |
| P2.1 | 0.40± 0.03defg | 0.37±0.06 efg | 0.48± 0.03bc | 0.48±0.02a | 0.27± 0.02ef | 0.26 ± 0.00e | 0.27± 0.12bd | 0.28±0.12b |
| P1.1 | 0.44± 0.05efg | 0.44±0.05efg | 0.43± 0.01ac | 0.50±0.02a | 0.27± 0.01ef | 0.26 ± 0.02e | 0.31± 0.13cd | 0.35±0.12c |

Note: Mean ± SE followed by different letters in the same column is significantly different at P = 0.05 by the Tukey simultaneous comparison test.
