# Supplementary material for: Growth, seed development and genetic analysis in wild type and Def mutant of Pisum sativum L
Source: BMC Res Notes. 2011 Nov 11;4:489. doi: 10.1186/1756-0500-4-489 (PMC3231984; doi:10.1186/1756-0500-4-489)
Supplement: Additional file 2 [file 1756-0500-4-489-S2.DOC]

**Additional file 2: Table 2**. R^2^ values for the relationship between width of funiculus (WFN) and other predictors.

|  |  | ***R*^2^** | | | |
| --- | --- | --- | --- | --- | --- |
| **Predictors** |  | **JI 116** | **JI 2822** | **JI 1184** | **JI 3020** |
| *Fresh Weight (FW)* |  | 19.6 | 57.4 | 51.7 | 55.9 |
| *Seed Width (SW)* |  | 14.8 | 49.0 | 48.2 | 53.2 |
| *Seed Height (SH)* |  | 17.9 | 49.5 | 53.5 | 56.6 |
| *FW+SW* |  | 21.1 | 59.2 | 51.8 | 57.3 |
| *FW+SH* |  | 19.6 | 57.2 | 55.0 | 62.7 |
| *SW + SH* |  | 18.2 | 55.2 | 54.9 | 65.3 |
| *FW+SW+SH* |  | 21.7 | 59.2 | 55.1 | 65.3 |
| *FW+SW+SH+FW*SH* |  | 24.2 | 59.2 | 54.4 | 66.3 |
| *FW+SW+H+FW*SW* |  | 26.2 | 59.5 | 55.2 | 68.7 |
| *FW+SW+SH+SW*SH* |  | 27.5 | 59.2 | 55.1 | 66.6 |
| *FW+SW+SH+FW*SW*SH* |  | 25.4 | 59.3 | 55.1 | 67.7 |
|  |  |  |  |  |  |

FW: Seed Fresh Weight

SH: Seed Height

SW: Seed Width

FW*SH: Interaction between Fresh Weight and Seed Height

FW*SW: Interaction between Fresh Weight and Seed Width

H*SW: Interaction between Height and Seed Width

FW*SH*SW: Interaction between Fresh Weight, Seed Height and Seed Width

Dependent variable: Width of Funiculus (WFN)
